# Best evidence summary on sexual health management for patients undergoing intracavitary brachytherapy for gynecological neoplasms

**DOI:** 10.3389/fonc.2026.1770806

**Published:** 2026-01-29

**Authors:** Rui Liu, Zhanxin Fan, Hailong Ma, Jianyan Ye, Liqing Chen, Meirong Qin, Lin Wang

**Affiliations:** 1 Department of Abdominal Radiation Oncology, Zhongshan People’s Hospital, Zhongshan, China; 2 College of Nursing and Health, Henan University, Kaifeng, China; 3 Nursing Department, Zhongshan People’s Hospital, Zhongshan, China

**Keywords:** brachytherapy, evidence summary, gynecologic oncology, sexual health, uterine cervical neoplasms

## Abstract

**Objective:**

To systematically retrieve, appraise and synthesize the best available evidence on sexual health management in patients undergoing intracavitary brachytherapy for cervical cancer, so as to provide an evidence-based foundation for developing individualized sexual-health care plans in clinical practice.

**Methods:**

A comprehensive computer-based search of domestic and foreign databases, guideline repositories and professional association websites was conducted for all evidence on sexual health management in cervical-cancer patients receiving intracavitary brachytherapy. Document types included guidelines, evidence summaries, systematic reviews, expert consensus statements and clinical decision aids. The search timeframe spanned database inception to June 2025.

**Results:**

Twelve publications were ultimately included: two clinical decision aids, one guideline, four systematic reviews, two evidence summaries, one expert consensus and two randomized controlled trials. Thirty-nine evidence statements were extracted and grouped under seven themes: target population and risk factors, screening and assessment, health education, non-pharmacologic interventions, pharmacologic and hormonal therapies, special treatments, and follow-up.

**Conclusion:**

This study summarizes the best current evidence on sexual health management for cervical-cancer patients undergoing intracavitary brachytherapy and offers valuable guidance for improving patients’ quality of life and sexual-health outcomes.

**Systematic Review Registration:**

http://ebn.nursing.fudan.edu.cn/home, identifier ES20256976.

## 1 Introduction

In recent years, the incidence of gynecological tumors in China has been increasing annually. In 2022, there were approximately 290,000 new cases of gynecological tumors and about 100,000 deaths (1). The three most common gynecological tumors by incidence are cervical cancer, endometrial cancer, and ovarian cancer (2). Most patients are diagnosed at an advanced stage, and radiotherapy and chemotherapy are often employed clinically (3). For locally advanced gynecological tumors, the traditional standard strategy involves external beam radiotherapy (EBRT) combined with concurrent chemotherapy, followed by intracavitary brachytherapy (ICBT) (4). Intracavitary brachytherapy (ICBT), also known as intracavitary afterloading radiotherapy, is a radiation technique that involves placing specialized applicators through natural cavities such as the vagina or uterus, followed by computer-guided precise delivery of radioactive sources to the tumor target area. As one of the mainstream modern radiotherapy techniques, ICBT is widely used in clinical practice and serves as an indispensable component of radical radiotherapy for cervical cancer (5). Existing studies indicate that radiotherapy for cervical cancer can cause significant vaginal mucosal injury and functional impairment, with the incidence of vaginal stenosis reaching as high as 88% (6). High-dose-rate afterloading therapy is prone to inducing stenosis of the cervical os and cervical canal, which may prevent the normal discharge of uterine fluid, clinically manifesting as characteristic lower abdominal distension and pain (7). These anatomical alterations not only compromise treatment efficacy but also exert multiple, long-lasting negative effects on quality of life. Sexual health is a broad construct that encompasses any diagnosis- or therapy-related dysfunction of sexual response, reproductive pelvic pain, or penetration difficulties. Such impairment arises from the interplay of biological, psychological and social factors; this multidimensional pattern of harm challenges existing one-dimensional interventions and calls for an evidence-based, integrated management program (8). Therefore, using rigorous evidence-based methodology, we systematically searched, critically appraised and synthesized the available evidence to provide guidance for the sexual-health management of cervical-cancer patients undergoing intracavitary brachytherapy, with the ultimate goal of improving survivors’ quality of life. The review protocol was prospectively registered with the Evidence-Based Nursing Center of Fudan University (registration number: ES20256976).

## 2 Information and methods

### 2.1 Establishment of evidence-based issues

Based on the “PIPOST” model established by the Fudan University Center for Evidence-Based Nursing, the evidence-based question was structured as follows (9): P (Population): Patients receiving intracavitary brachytherapy for cervical cancer; I (Intervention): Sexual health management strategies;

P (Professional): Evidence users, including patients, healthcare professionals, and family caregivers;

O (Outcome): Overall sexual function status of patients;

S (Setting): Gynecological oncology wards, outpatient clinics, and home-based environments;

T (Type of evidence): Guidelines, evidence summaries, expert consensus, clinical decisions, practice recommendations, systematic reviews, and randomized controlled trials.

### 2.2 Literature search strategy

**Figure 1:**
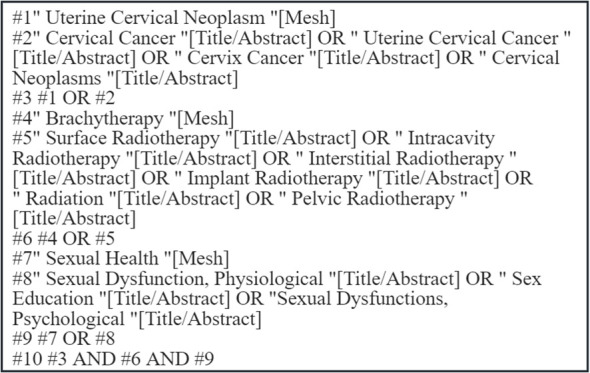
PubMed search strategy.

Following the top-down search principle based on the “6S” evidence pyramid model (10), we systematically searched the following databases: UpToDate, Cochrane Library, CINAHL, PubMed, Embase, Web of Science, Wanfang Data, CNKI (China National Knowledge Infrastructure), CBM (Chinese Biomedical Literature Database), and VIP Database. Additionally, the following guideline and professional organization websites were consulted: the Guidelines International Network (GIN), National Comprehensive Cancer Network (NCCN), Canadian Medical Practice Guidelines Database, National Institute for Health and Care Excellence (NICE), Cancer Care Ontario (CCO), American Society of Clinical Oncology (ASCO), American Society for Radiation Oncology (ASTRO), European Society of Gynaecological Oncology (ESGO), European Society for Medical Oncology (ESMO), and Medlive Guideline Network. Using search terms including “Uterine Cervical Neoplasm/Cervical Cancer/Uterine Cervical Cancer/Cervix Cancer/Cervical Neoplasms”, “Radiation/Pelvic Radiotherapy/Brachytherapy/Surface Radiotherapy/Intracavity Radiotherapy/Interstitial Radiotherapy/Implant Radiotherapy”, and “Sexual Health/Sexual Dysfunction, Physiological/Sex Education/Sexual Dysfunctions, Psychological”, a combined search of subject headings and free-text terms was conducted. The search period covered records from the inception of each database to June 2025. The specific search strategy for PubMed is illustrated in **Figure 1**.

### 2.3 Literature inclusion and exclusion criteria

Inclusion Criteria: The study content pertained to sexual health management strategies. Study types included: guidelines, expert consensus, evidence summaries, clinical decision support documents, clinical practice recommendations, systematic reviews, and randomized controlled trials. Publications were limited to those in Chinese or English. Exclusion Criteria: Articles with incomplete or missing information. Studies for which the full text was unavailable. Duplicate publications. Studies that did not pass the literature quality assessment.

### 2.4 Literature quality evaluation criteria

The guideline appraisal process was independently conducted by a panel of four experts using the Appraisal of Guidelines for Research and Evaluation II (AGREE II) instrument (11).

For other types of literature, the quality assessment was conducted independently by two researchers with expertise in evidence-based methodology. They systematically reviewed all included studies and evaluated their quality. Any disagreements in assessment were resolved through discussion or by consulting a third reviewer. Specific appraisal tools were applied as follows: systematic reviews and expert consensus documents were evaluated using the Joanna Briggs Institute (JBI) Critical Appraisal Tools (2016 version) (10); randomized controlled trials were assessed with the Cochrane Risk of Bias Tool (12); for evidence summaries and clinical decision support documents, quality appraisal was performed by tracing back to the original source evidence and applying the appropriate tool based on the study design.

### 2.5 Evidence extraction, synthesis, and evaluation

Two researchers independently extracted evidence from the included literature. Following extraction, the evidence was translated, cross-checked, and synthesized. In cases of conflicting conclusions from different sources, this study adhered to a hierarchy prioritizing evidence-based sources, high-quality studies, the most recently published authoritative literature, and domestic guidelines. The integrated results were verified by a third researcher. Using the JBI Evidence Pre-grading and Recommendation Level System (2014 version) (13), the original studies of the finally included evidence were graded from Level 1 (highest) to Level 5 (lowest). The recommendation strength for each piece of evidence was determined based on its FAME attributes (Feasibility, Appropriateness, Meaningfulness, Effectiveness), with Grade A representing strong recommendation and Grade B weak recommendation.

## 3 Results

### 3.1 General characteristics of the included literature

**Figure 2:**
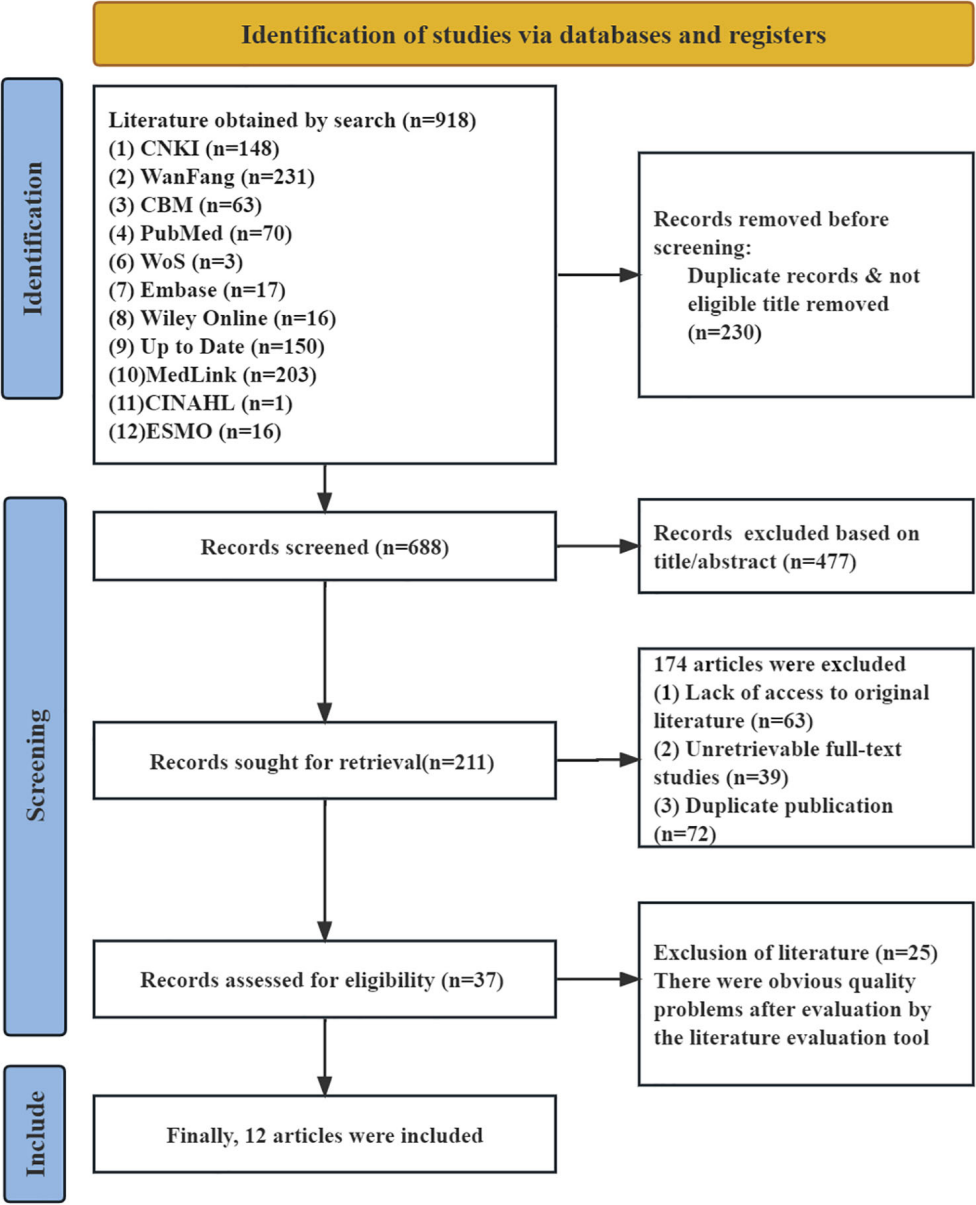
Flow diagram of literature search.

A total of 918 publications were retrieved. After removing duplicates using EndNote and manual screening, reviewing titles and abstracts for preliminary screening, and assessing full texts for final eligibility, 12 articles were ultimately included. These comprised 2 clinical decision support documents (14, 15), 1 guideline (16), 1 expert consensus (17), 4 systematic reviews (18–21), 2 evidence summaries (22, 23), and 2 randomized controlled trials (24, 25). The literature search flow diagram is presented in **Figure 2**, and the general characteristics of the included studies are summarized in **Table 1**.

**Table 1:** Basic characteristics of included studies(n=12).

Producer/Author	Publication year	Literature source	Type of evidence	Literature topics
Martins et al. (24)	2021	WOS	RCT	Prevention of vaginal stenosis in cervical cancer patients after radiotherapy using local estrogen, testosterone, and vaginal dilators
Suvaal et al. (25)	2024	PubMed	RCT	Efficacy of nurse-led sexual rehabilitation interventions for gynecological cancer patients undergoing radiotherapy
Cibula et al. (16)	2023	PubMed	Guideline	Guidelines for the management of cervical cancer patients
Matos et al. (17)	2019	CINAHL	Expert Consensus	Consensus on prevention of vaginal stenosis in patients undergoing radiotherapy
Tramacere et al. (18)	2022	PubMed	Systematic Review	Systematic review of sexual dysfunction in cervical cancer patients following different treatment modalities
Cianci et al. (19)	2023	PubMed	Systematic Review	Systematic review on sexual function and quality of life in cervical cancer patients post-treatment
Ye et al. (20)	2014	PubMed	Systematic Review	Systematic review of quality of life and sexual function in cervical cancer survivors
Wiltink et al. (21)	2020	PubMed	Systematic Review	Impact of contemporary cervical cancer treatment on patient-reported health-related quality of life: a systematic review
Dai Qiongfang et al. (22)	2024	CNKI	Evidence Summary	Best evidence summary for sexual health management in cervical cancer patients
Li Songlan et al. (23)	2024	CNKI	Evidence Summary	Evidence summary on vaginal dilation rehabilitation for cervical cancer radiotherapy patients
Lindard et al. (14)	2022	Up to Date	Clinical Decision-Making	Comprehensive overview of care approaches for cervical cancer survivors
Reed et al. (15)	2022	Up to Date	Clinical Decision-Making	Overview of female sexual dysfunction

### 3.2 Quality evaluation results of the included literature

#### 3.2.1 Quality evaluation results of the guidelines

This study included one guideline (16). As all six domain standardized scores of this guideline were ≥60%, it was assigned a Grade A recommendation. The appraisal results are summarized in **Table 2**.

**Table 2:** Results of the quality evaluation of the guidelines.

Inclusion guidelines	Standardized scores in various domains (%)					Editorial independence	≥ 60% Field number(n)	≥30% Field number (n)	Recommendation level
	Scope and purpose	Involved personnel	The rigor of the guidelines	Clarity of guidelines	Applicability				
Cibula et al. (16)	83.3	90.7	91.7	94.4	83.3	100	6	0	A

#### 3.2.2 Quality evaluation results of the systematic review

Four systematic reviews were included (18–21). The critical appraisal results indicated that three reviews (19–21) were rated “unclear” on Item 9 (assessment of publication bias) but “yes” on all other items. The remaining review (18) was rated “unclear” on Item 7 (measures to minimize errors in data extraction) and “yes” on all other items. Detailed appraisal results are presented in **Table 3**.

**Table 3:** Quality evaluation results of the systematic review.

Systematic reviews inclusion and evaluation	Evaluation items											Inclusion (Yes/No)
	①	②	③	④	⑤	⑥	⑦	⑧	⑨	⑩	⑪	
Tramacere et al. (18)	yes	yes	yes	yes	yes	yes	unclear	yes	yes	yes	yes	yes
Cianci et al. (19)	yes	yes	yes	yes	yes	yes	yes	yes	unclear	yes	yes	yes
Ye et al. (20)	yes	yes	yes	yes	yes	yes	yes	yes	unclear	yes	yes	yes
Wiltink et al. (21).	yes	yes	yes	yes	yes	yes	yes	yes	unclear	yes	yes	yes

①Is the evidence-based question raised clear and explicit? ②Is the inclusion criteria for literature appropriate for this evidence-based question? ③Is the retrieval strategy appropriate? ④Is the search database or resources sufficient? ⑤Is the literature quality evaluation standard used appropriately? ⑥Are there 2 or more evaluators independently completing quality evaluations? ⑦Are certain measures taken to reduce errors when extracting data? ⑧Is the method of merging research appropriate? ⑨Has the possibility of publication bias been evaluated? ⑩Are the policy or practice recommendations based on the results of a systematic evaluation? ⑪Is the proposed further research direction appropriate?

#### 3.2.3 Quality evaluation results of clinical decision-making and evidence summarization

This study included two clinical decision support documents (14, 15) and two evidence summaries (22, 23). The original studies traced for these documents comprised two randomized controlled trials (RCTs) (24, 25), one guideline (16), and one systematic review (18). All original studies underwent corresponding quality appraisal and were subsequently included.

#### 3.2.4 Quality evaluation results of expert consensus

One expert consensus document was included (17), which received a “yes” rating on all appraisal items.

#### 3.2.5 Quality evaluation results of randomized controlled studies

Two randomized controlled trials (RCTs) were included in this study (24, 25). Apart from the items concerning “blinding of investigators and participants” and “blinding of outcome assessors,” which were rated “no,” all other items in both studies received a “yes” rating. Given their overall high quality, the studies were included.

### 3.3 Summary of evidence

A preliminary extraction yielded 39 relevant pieces of evidence. Through team analysis, comparison, and discussion, content with similar themes was categorized and synthesized. This process ultimately formed seven thematic categories: applicable populations and risk factors, screening and assessment, health education, non-pharmacological interventions, pharmacological and hormonal therapy, specialized treatments, and follow-up care, as presented in **Table 4**.

**Table 4:** Literature extraction.

Evidence items	Evidence content	Level of evidence	Recommended level
Applicable Populations and Risk Factors	1. Patients who were sexually active prior to treatment; individuals who underwent intracavitary brachytherapy for cervical cancer combined with external beam radiotherapy (18).	Level5	B
	2. Associated risk factors include: ① Disease-related factors: Radical hysterectomy and pelvic radiotherapy, with cervical cancer patients receiving radiotherapy being more prone to developing sexual dysfunction of longer duration compared to those undergoing surgery or chemotherapy alone;② Treatment-related sequelae: Dyspareunia, pelvic floor dysfunction, genitourinary syndrome of menopause, and persistent fatigue;③ Psychological factors: Body image disturbances, intimacy issues, and depression (14, 19–22).	Level2	A
Screening and Assessment	3. Screening and counseling for sexual dysfunction at any time point before, during, or after treatment (14, 22, 24, 25)	Level4	A
	4. It is recommended to use simple universal inquiries to inform, confirm, and ask related questions (14, 15, 22).	Level5	B
	5. Comprehensive diagnostic evaluation includes: ① Medical history: the patient's complete medical history, gynecological history, and full cancer treatment history; ② Sexual history: early sexual behavior history or history of sexual trauma; ③ Physical examination: general physical examination and gynecological examination (14, 15, 22).	Level5	A
	6. Simple screening tools, such as the Female Cancer Patients' Sexual Function Symptom Questionnaire, can be used to guide clinical inquiries (14).	Level1	A
	7. Currently, the scale tools available in China for assessing the sexual function of cervical cancer patients include the Female Sexual Function Index (FSFI), the European Organization for Research and Treatment of Cancer Quality of Life Questionnaire for Cervical Cancer Patients (EORTC QLQ-CX24), the Patient-Reported Outcomes Measurement Information System (PROMIS-SexFS), the Functional Assessment of Cancer Therapy-Cervix (FACT-Cx), and the Sexual Activity-Vaginal Changes Questionnaire (SVQ) (14, 17, 20–22).	Level4	A
Health Education	8. Provide two types of patient education materials: "Basic Version" and "Advanced Version." The Basic Version is easy to understand and suitable for patients who want an overview of the disease and prefer concise, readable materials. The Advanced Version is longer and more detailed, with in-depth content suitable for patients who wish to gain a deeper understanding and can handle some medical terminology (14).	Level5	B
	9. Explain to patients that vaginal toxicity can adversely affect their quality of life (22).	Level5	A
	10. Educate patients on vulvovaginal anatomy and the pelvic floor to help them understand the mechanisms and causes of genito-pelvic pain and penetration disorders. Instruct patients to avoid vulvovaginal contact with irritants, including soap, douches, wipes, scented products, and panty liners (15).	Level3	A
Non-pharmacological Intervention	11. A multidisciplinary team should be established, primarily comprising oncology specialist nurses, radiation oncologists, gynecologic oncologists, psychotherapists, and sexologists (18, 23), to provide patients with comprehensive management throughout the vaginal dilation rehabilitation process.	Level3	A
	12. Dedicated healthcare professionals should consistently provide information on vaginal dilation. It is recommended that the initial explanation be given by a radiation oncologist, followed by continuous support from a designated oncology nurse who will offer information, psychological, and practical assistance throughout the rehabilitation period (18, 23).	Level1	B
	13. The selection of dilator types can be personalized and may include penile prostheses, silicone or plastic dilators, and other materials suitable for the vaginal area (18, 23).	Level5	A
	14. A condom can be used to cover the vaginal dilator during insertion, and an adequate amount of lubricant should be applied to reduce discomfort (23).	Level1	B
	15. Use a flat-tipped dilator, starting with a diameter smaller than that of the vagina, and gradually transition to dilators with larger circumferences until a comfortable diameter/appropriate vaginal size is achieved (18, 23).	Level5	A
	16. If a woman wishes to preserve vaginal function after brachytherapy, vaginal dilators should be used within 2–4 weeks after treatment completion. A lubricated vaginal dilator should be inserted into the vagina for 10–20 minutes, three times per week. The adjunctive use of vaginal lubricants, moisturizers, or topical hormonal agents prior to dilation can help reduce vaginal bleeding. It is recommended to continue using the dilator for at least one year after radiotherapy (14, 16, 18, 19, 22).	Level3	B
	17. The depth and position of insertion should be determined by the patient. The dilator should be inserted as deeply as possible (reaching the top of the vagina to a comfortable position) and maintained for a certain period, then gently rotated before removal. During insertion, movements should be moderate to avoid damaging the vaginal mucosa (18, 23).	Level5	B
	18. To maximize the effectiveness of vaginal dilators, patients should be referred to a pelvic floor physiotherapist for demonstration and instruction on the correct usage of the device (14).	Level5	B
	19. Under the guidance of a pelvic floor physiotherapist, patients may engage in pelvic floor muscle massage, pelvic floor muscle exercises (such as Kegel exercises, yoga, and core functional training), pelvic floor electrical stimulation therapy (including transcutaneous electrical nerve stimulation, TENS), and intravaginal physiotherapy (14, 15, 19, 22, 23, 25).	Level3	A
	20. Vaginal dilation or sexual activity should not be performed during or immediately after radiotherapy (23).	Level1	B
	21. If the patient resumes normal sexual activity, the frequency of vaginal dilator use can be reduced (18, 23).	Level5	B
	22. For individuals without sexual activity, indefinite use of a dilator is recommended (23).	Level5	A
	23. Sexual aids (e.g., vibrators) may be used, and patients should also be advised to employ lubricated sexual aids (14, 15, 18, 19, 22).	Level5	A
	24. Sexual activity can be resumed when the patient feels comfortable, ideally 2–4 weeks after radiotherapy, once the vaginal mucosa has recovered (23).	Level1	A
	25. For patients experiencing decreased libido, cognitive behavioral therapy, mindfulness-based therapies, and couple therapy should be incorporated as part of the treatment for female sexual dysfunction (14, 15, 22, 25).	Level1	A
	26. Referral to or consultation with mental health professionals who possess specialized knowledge and training in the treatment of female sexual dysfunction should be considered based on the clinician's expertise and the patient's individual therapeutic needs (14, 15, 18, 20, 23).	Level3	A
	27. For female patients who explicitly express concerns about body image, psychological issues, or relationship difficulties, referral for sex therapy or couples psychotherapy is also recommended (14).	Level5	B
Pharmacological and Hormonal Therapy	28. Personal Lubricants — Generally fall into three categories: water-based, silicone-based, and oil-based. They are suitable for use during intercourse to alleviate discomfort and friction caused by sexual activity or vaginal insertion. Vulvovaginal Moisturizers — Include vaginal moisturizing creams, pH-balancing gels, hyaluronic acid suppositories, vitamin E suppositories, and natural oils, among others (14, 19, 22, 25).	Level5	A
	29. For some female cancer survivors who do not experience relief from dyspareunia with non-hormonal treatments, hormonal therapy may be considered as a subsequent option. For instance, low-dose estrogen can be applied to the vulvovaginal area. Current evidence suggests that locally administered low-dose estrogen or estradiol to the vaginal introitus and/or vagina is most effective in treating dyspareunia caused by genitourinary syndrome of menopause (14–16, 19, 22, 23).	Level1	A
	30. In the absence of contraindications, estrogen therapy initiated within 6 months after radiotherapy (typically starting between 3 to 6 months) can improve vaginal symptoms and reduce the incidence of vaginal stenosis (23, 24).	Level1	B
	31. Ospemifene can improve sexual issues in postmenopausal women caused by genitourinary syndrome (15).	Level1	B
	32. Flibanserin may be used as a treatment for decreased sexual desire in premenopausal women without depression. Its use requires alcohol abstinence and should be conducted under medical supervision (15).	Level1	B
	33. Bupropion may be used to treat sexual side effects caused by antidepressant medications (15).	Level1	A
	34. Vaginal suppositories containing hyaluronidase, vitamin A, and vitamin E can be used twice daily (in the morning and before bedtime) for a duration of four months. They may be administered concurrently with radiotherapy during the first two months to help reduce vaginal side effects (23).	Level1	B
Specialized Therapies	35. Vaginal laser therapy can improve vaginal dryness, dyspareunia, and enhance sexual function. Treatments are administered once every 4–6 weeks, typically for a total of 3 sessions within a specified period (14, 19, 22).	Level3	B
	36. Fertility preservation measures include embryo cryopreservation, oocyte cryopreservation, ovarian tissue cryopreservation, fertility-sparing radical trachelectomy, and ovarian transposition for patients undergoing pelvic radiotherapy (16, 22).	Level3	B
	37. Patients who have undergone fertility-sparing surgery are at high risk for pregnancy complications. Maternal-fetal medicine specialists should be involved in their management, and pregnancy monitoring should be intensified (16, 22).	Level3	B
Follow-up	38. An additional 30-minute follow-up/telephone consultation may be scheduled between 6 to 12 months after treatment (25).	Level1	A
	39. The use of vaginal dilators should be monitored during each follow-up consultation (18).	Level1	A

## 4 Discussion

### 4.1 Comprehensiveness and systematicity of the evidence system

The most significant value of this evidence summary lies in its systematic and continuum-based perspective. Traditionally, attention to post-radiotherapy sexual health has often focused narrowly on “treatment” after symptom onset, such as the use of lubricants or estrogen (26). In contrast, the evidence framework constructed in this study extends forward to risk identification and screening assessment, backward to long-term follow-up, and emphasizes the central role of preventive health education. This demonstrates that effective sexual health management is not an isolated intervention point, but rather a continuous and dynamic process that spans the pre-, during-, and post-radiotherapy phases. The integrated application of the seven major themes aligns with the modern oncology rehabilitation concept of “comprehensive and whole-cycle” management, providing a solid theoretical framework for developing standardized clinical management pathways.

### 4.2 In-depth analysis and clinical implications of evidence across themes

#### 4.2.1 Applicable populations and risk factors

The evidence clearly indicates that not all patients face the same level of sexual health risk, underscoring the importance of risk stratification in this population. In clinical practice, high-risk individuals should be identified early and provided with more intensive screening and preventive health education resources. This approach enables optimized resource allocation and serves as an initial step toward personalized management (27, 28).

#### 4.2.2 Screening and assessment

Sexual health issues are often highly private and stigmatized, and patients may actively conceal them (29, 30). Sexual health assessments should be integrated throughout the entire course of three-dimensional brachytherapy for cervical cancer patients, with screening and counseling for sexual dysfunction conducted during the pre-treatment, during treatment, and post-treatment phases (14, 22, 24, 25). When implementing screening, it is recommended to use brief, universal inquiries as a starting point to inform, confirm, and preliminarily understand potential issues the patient may have (14, 15, 22). If problems are identified or further evaluation is needed, a comprehensive diagnostic assessment should be initiated, covering medical history, sexual history, and physical examination (14, 15, 22).

Regarding the application of screening tools, brief instruments (such as the Sexual Function-Vaginal Changes Questionnaire for patients with cervical cancer) can be used to guide initial clinical inquiries (14). For cervical cancer patients, commonly used specialized assessment tools currently include the Female Sexual Function Index (FSFI), the European Organization for Research and Treatment of Cancer Quality of Life Questionnaire Cervical Cancer Module (EORTC QLQ-CX24), the Patient-Reported Outcomes Measurement Information System Sexual Function and Satisfaction measure (PROMIS-SexFS), the Functional Assessment of Cancer Therapy-Cervix (FACT-Cx), and the Sexual Activity-Vaginal Changes Questionnaire (SVQ), among others (14, 17, 20–22). Currently, there is a wide variety of sexual assessment scales for cervical cancer, each with different application purposes and characteristics, and no single standard is available to evaluate all these scales (31).

#### 4.2.3 Health education

The evidence underscores the importance of health education. Many cervical cancer patients, lacking timely and effective sexual health guidance, actively avoid sexual activity due to fears that it may cause tumor recurrence or transmission after treatment. Simultaneously, their spouses often become overprotective toward the patient or themselves (32). Healthcare providers can offer patients two types of educational materials: “Basic Edition” and “Advanced Edition” (14). These materials explain how vaginal toxicity can adversely affect the patient’s quality of life (22) and guide patients in avoiding irritants that come into contact with the vulva and vagina (15). This approach helps reduce patient anxiety and fear while enhancing their confidence and ability in self-management, which is key to achieving patient empowerment. In line with current internet trends and developments in healthcare informatization, there is an opportunity to actively develop new models of sexual health education based on “Internet + Nursing Services” (33). Currently, the “Internet + Nursing Services” model has been widely promoted and applied in areas such as venous catheter maintenance and wound and stoma care (34).

#### 4.2.4 Non-pharmacological and pharmacological/hormonal interventions

This evidence summary categorizes interventional measures into two major types: non-pharmacological and pharmacological/hormonal therapies, establishing a comprehensive intervention system supported by a multidisciplinary team and centered on vaginal dilation therapy. It emphasizes the provision of continuous guidance by designated healthcare professionals (e.g., oncology specialist nurses), covering aspects such as treatment timing (initiating 2–4 weeks after radiotherapy), frequency (three times per week for at least one year), and specific procedures (starting with small sizes, ensuring adequate lubrication, and gentle insertion). Lubricants and moisturizers play a crucial supportive role in this process: personal lubricants (water-based, silicone-based, or oil-based) can provide immediate relief during intercourse or dilation, while vulvovaginal moisturizers (such as suppositories or creams containing hyaluronic acid or vitamin E) are used to improve vaginal moisture and tissue health over the long term (14, 19, 22, 25). Additionally, referrals to pelvic floor physical therapists should be made when necessary to enhance rehabilitation outcomes, and psychological interventions such as cognitive behavioral therapy, couples counseling, or psychosexual therapy should be integrated to address psychological distress (14).

When non-hormonal methods fail to adequately alleviate dyspareunia, pharmacological and hormonal therapies play a critical role (35, 36). Topical low-dose estrogen is considered one of the most effective approaches for managing dyspareunia caused by genitourinary syndrome of menopause (GSM). After contraindications are excluded, it may be initiated within six months post-radiotherapy (typically starting at 3–6 months) to improve vaginal symptoms and reduce the incidence of vaginal stenosis (14–16, 19, 22–24). Additionally, systemic medications such as ospemifene can be used to address GSM-related concerns in postmenopausal women (15–37). For hypoactive sexual desire disorder, flibanserin (suitable for premenopausal women without depression) and bupropion (particularly for antidepressant-induced sexual side effects) may be considered options (15). During and after radiotherapy, the use of vaginal suppositories containing hyaluronidase, vitamin A, and vitamin E (e.g., twice daily for four months, with the first two months concurrent with radiotherapy) has also been demonstrated to effectively reduce the occurrence of vaginal adverse reactions (23). It is crucial to emphasize that healthcare providers should proactively guide and encourage patients experiencing sexual health difficulties to seek professional help. Following standardized medication guidance for symptomatic treatment can help avoid the risks of incorrect medication use or drug misuse associated with self-directed online information searches.

#### 4.2.5 Specialized therapies and follow-up

Although the incidence of cervical cancer has declined, its trend toward younger onset deserves attention. According to statistics, approximately 40% of cervical cancer patients are diagnosed at reproductive age. Women of childbearing age diagnosed with cervical cancer often express a desire to preserve fertility (38). Fertility preservation can be achieved through methods such as embryo cryopreservation, oocyte cryopreservation, ovarian tissue cryopreservation, or fertility-sparing radical trachelectomy (16, 22). It is important to emphasize that patients undergoing such fertility-sparing procedures face a relatively higher risk of pregnancy-related complications. Therefore, it is strongly recommended that maternal-fetal medicine specialists be involved in the long-term management of these patients, with intensified monitoring throughout pregnancy to ensure the safety of both the mother and the child (16, 22).

Evidence on long-term follow-up emphasizes scheduling an additional 30-minute follow-up session or telephone consultation between 6 and 12 months after treatment (25). Concurrently, during each follow-up consultation, monitoring should include the usage of vaginal dilators and the occurrence of vaginal adverse reactions (18–39). Where medical resources permit, establishing a nurse-led pelvic health clinic within radiation oncology departments is recommended to provide patients with relevant consultations and health education (40).

### 4.3 Limitations and future directions

#### 4.3.1 Limitations

Firstly, the current number of high-quality randomized controlled trials in this field remains relatively limited, with some recommendations based on observational studies or expert consensus, indicating that the evidence base needs further strengthening. Secondly, as the integrated evidence is drawn from global literature, its application to clinical practice in specific countries or regions requires careful consideration of localization factors such as cultural differences, accessibility of medical resources, and health insurance policies. Additionally, from a methodological perspective, the search was limited to published academic literature and did not include grey literature (e.g., dissertations, conference proceedings, or clinical trial registry data). While this approach helps ensure the standardization and methodological quality of the included evidence, it may not fully reflect the latest research developments, introducing potential selection bias.

#### 4.3.2 Future directions

High-quality interventional studies should be developed, employing rigorous randomized controlled trials to validate the efficacy of combined interventions (e.g., health education with pelvic floor muscle training and vaginal dilators). Further investigation is needed to explore optimized multidisciplinary collaboration models involving oncologists, nurses, psychologists, and rehabilitation therapists in sexual health management. Future evidence synthesis ought to place greater emphasis on partner engagement and psychological well-being, incorporating strategies to encourage partner support and developing more profound interventions targeting psychosexual trauma. Additionally, future systematic reviews could further expand the search scope to incorporate more grey literature, thereby constructing a more comprehensive evidence system.

## 5 Conclusion

In summary, this study systematically synthesizes evidence across seven thematic areas, collectively establishing a scientific, comprehensive, and practical framework for sexual health management in cervical cancer patients undergoing three-dimensional brachytherapy. The framework emphasizes patient-centered care and implements holistic, multidimensional management spanning from risk prevention to long-term follow-up. Translating this evidence system into clinical practice holds significant potential to improve patients’ sexual health outcomes and enhance their long-term quality of life, representing an indispensable component of cervical cancer rehabilitation.

## Data Availability

The original contributions presented in the study are included in the article/supplementary material. Further inquiries can be directed to the corresponding author.
